# Profiles of vulnerability for suicide and self-harm in UK prisoners: Neurodisability, mood disturbance, substance use, and bullying

**DOI:** 10.1371/journal.pone.0296078

**Published:** 2024-01-03

**Authors:** Hope Kent, Bella Magner-Parsons, George Leckie, Tuna Dulgar, Anggita Lusiandari, Lee Hogarth, Huw Williams, Amanda Kirby

**Affiliations:** 1 Department of Psychology, Washington Singer Laboratories, University of Exeter, Devon, United Kingdom; 2 School of Education, University of Bristol, Bristol, United Kingdom; 3 Emeritus Professor, School of Education, University of South Wales, Wales, United Kingdom; Virginia Tech Carilion School of Medicine, UNITED STATES

## Abstract

Screening for vulnerability factors associated with historic suicidality and self-harm on entry to prison is critical to help prisons understand how to allocate extremely limited mental health resources. It has been established that having previous suicide attempts increases odds of future suicidality and self-harm in prison. We utilised administrative screening data from 665 adult male prisoners on entry to a category B prison in Wales, UK, collected using the Do-IT Profiler. This sample represents 16% of all prisoners who entered that prison during a 26-month period. 12% of prisoners reported a history of attempted suicide, 11% reported historic self-harm, and 8% reported a history of both. Historic traumatic brain injury and substance use problems were associated with a 3.3- and 1.9- times increased odds of a historic suicide attempt, respectively, but no significant increased risk of historic self-harm (95% CI: 1.51–6.60 and 1.02–3.50). However, those who were bullied at school had 2.7 times increased odds of reporting a history of self-harm (95% CI: 1.63–6.09). The most salient risk factors associated with *both* historic suicide and self-harm were higher levels of functional neurodisability (odds ratio 0.6 for a 1 standard deviation change in score, 95% CI: 0.35–0.75), and mood disturbance (odds ratio 2.1 for a 1 standard deviation change in score, 95% CI: 1.26–3.56). Therefore, it could be beneficial for prisons to screen for broader profiles of needs, to better understand how to provide appropriate services to prisoners vulnerable to suicide and self-harm. Multidisciplinary care pathways for prisoner mental health interventions are important, to account for complex multimorbidity. Adaptations may be needed for mental health interventions to be appropriate for, for example, a prisoner with a brain injury. Understanding this broad profile of vulnerability could also contribute to more compassionate responses to suicide and self-harm from prison staff.

## Introduction

It is critical that prisons understand the mental health needs of prisoners at the point of entry to prison, to allocate their limited psychiatric resources to the people who need them most and design care pathways that account for the needs of prisoners who use them. It has been established that having historical suicide attempts increases an individual’s odds of a suicide attempt in prison, with a recent meta-analysis finding an odds ratio of 8.2 [1]. Historic self-harm is also an established risk factor for self-harm in prison, and prisoner self-harm is related to subsequent suicide in prison [2]. However, a report from the House of Commons Justice Committee [3] found that mental health resources are seriously inadequate, with only around 10% of prisoners in the UK receiving interventions for mental health at any one time whilst an estimated 70% are in need at any one time. Ultimately, there is serious under-resourcing of prison mental health care [4]. As well as acute distress for incarcerated people and their families, prisoner suicide and self-harm also has a critically harmful impact on the wellbeing of prison staff [5,6]. To address these issues, this study utilised administrative screening data from adult male prisoners on entry to a category B prison in Wales, UK to determine the individual risk factors most associated with reporting historic suicide attempts and self-harm on entry to prison, in order to characterise a broader profile of vulnerability which might be used to inform allocation of limited mental health resources in prisons. The wider benefits of this are twofold. Firstly that an understanding of a broader range of risk factors could inform the development of care pathways for prisoners, particularly where multimorbidity may impact interventions. The second benefit of this approach is developing more compassionate responses within prison staff, in helping to offer a profile of vulnerability of individuals and demonstrating that suicide and self-harm are situated within a broader profile of multiple disadvantages.

### Prisoner suicide and self-harm

Suicides constitute 24% of deaths in criminal justice settings [7]. In a study of 24 European countries, prisoner suicide rates were estimated to be between 3- and 8-times higher than in the general population for males, and ten times higher for females, with many countries having absolute prisoner suicide rates of 100 per 100,000 prisoners [8]. Witnessing or being proximal to prisoner suicide can have a transformative effect on prison staff, with psychological impacts including distress, grief, shame, desensitisation, and PTSD, and a culture of stigmatisation meaning that officers are unlikely to seek support [6]. These traumatic experiences can impact staff behaviour, with staff becoming disengaged and emotionally distant, and contribute to vicious cycles where staff have an impaired ability to support prisoners in mental health crises [5]. Additionally, risk of compassion fatigue and burnout in prison staff is elevated by multiple exposure to traumatic experiences such as witnessing suicide fatalities and attempts [9].

The annual prevalence of self-harm in prisons is estimated at 5–6% of adult male prisoners and 20–24% of adult female prisoners [2]. Comparatively, annual prevalence of self-harm in the general population is estimated at 1% [10]. Half of prisoners who die by suicide in prison have a history of self-harm, which is associated with between six- and eleven-times increased odds of suicide [11]. A recent systematic review and mixed-methods synthesis found that prison officers often underestimate the association between self-harm and suicide [12], even though prior self-harm consistently predicts subsequent suicide in prison or after release [13]. Prison staff have been found instead to characterise self-harm as being manipulative or attention seeking, and to perceive institutional guidance on the appropriate responses to self-harm as being ambiguous [14].

Improving screening tools can be beneficial for enabling prisoners to access a higher quality of mental health care and reducing the emotional burden of prison staff [15,16]. Currently, in the USA, whilst it is mandated to screen for mental health problems, there is no standardised screening measure. This leads to variation in the questions asked, with evidence suggesting that some formats are ineffective [17]. In the UK, there is also significant discrepancy in the screening procedure, training of prison staff undertaking screening, and screening tools used, and prisoners with mental health problems are frequently missed in their first 24 hours in prison [18]. This is important, as 32% of prisoner suicides occur within the first 7 days in prison [19]. It is critical therefore to improve prison screening practices, and to be able to identify which risk factors are most important for inclusion in initial mental health assessments [20].

### Risk factors

We utilised data from the prison’s usual screening tool here, which included variables which have been previously linked to an elevated risk of self-harm and/or suicide in prisons or in the general population. These included a mental health questionnaire [21]; Traumatic Brain Injury (TBI) [22,23]; being bullied at school [24]; being excluded from school [25]; being homeless or marginally housed prior to prison [26]; being a veteran [27]; and current substance use problems [28]. A functional measure of neurodisability was also included. Neurodisability is an umbrella term, referring to a collection of congenital and acquired conditions affecting the brain and nervous system. These include, but aren’t limited to: Autism, attention-deficit hyperactivity-disorder (ADHD), learning disabilities, developmental co-ordination disorder, TBI, and foetal alcohol spectrum disorder (FASD). These conditions generally lead to functional deficits in one or more of the following domains: Cognitive, social/emotional, behavioural, and physical. Neurodisability has been associated with elevated risk of self-harm and suicide in the general population and in prisons. These factors are frequently comorbid, and comorbidity exacerbates risk. For example, children with TBI have been shown to be at increased odds of being bullied at school, and also at increased odds of attempted suicide [29,30]. Additional vulnerability is conferred when neurodisability is not understood in prisons, and behaviour associated with neurodisability is interpreted as defiance towards prison rules, which lead to exclusion from rehabilitative programs and removal of support [31]. Finally, elderly prisoners may also be at elevated risk of suicide in prison. This is hypothesised to be a result of a complex comorbid combination of mental and physical health needs, and victimisation within prisons [32]. We included age as a risk factor in this study.

### The current study

This study utilised administrative data to answer the following research question–‘What individual risk factors are uniquely associated with having historical suicide attempts and historic self-harm amongst adult male prisoners on entry to prison?’. Whilst some studies have examined the relationship between individual risk factors and subsequent suicide or self-harm in prisoners, no studies to our knowledge have examined the relationships between a broad matrix of risk factors and having historic suicide and self-harm on entry to prison. Our focus is on screening on entry to prison, as screening provides a unique opportunity to identify risk and implement preventative interventions, rather than reacting to mental health crises after they come to the attention of prison staff. It is important to note at this stage, that the administrative screening data were cross-sectional, and no temporal or contextual information was collected about historic suicidality or self-harm. As a result, we present our findings as cross-sectional associations, and do not propose causality.

## Method

### Data

The study examined administrative data from 852 adult male prisoners screened on entry to a category B prison in Wales, UK, between February 2014 and March 2016. The data were first accessed for research purposes in December 2021. In March 2016, the prison changed their screening procedures for mental health, so this is the most recent available data which included mental health screening. Some delay in accessing the data followed because of the operational challenges involved in accessing prison data for research purposes.

100 prisoners were being held on remand, meaning that they were being held pre-trial and had not been formally convicted of a crime at the time of screening, and 752 had been convicted. Table 1 describes the sample. Individuals were screened at induction to prison, with the option for people to decline if they did not wish to complete the screening. The prison receives approximately 1,900 new entrants per year, so we estimate that over the 26 month period between February 2014 and March 2016 our sample comprises approximately 21% of all new entrants over that time period. A STROBE (strengthening the reporting of observational studies in epidemiology) checklist for this study is available in the appendix.

**Table 1:** Frequency table describing n (with %) or mean with standard deviation for all variables.

Variable			Sample n = 852			
			N = (%)	Mean (SD)	Range	Missing n = (%)
Mental Health Symptoms	Psychosomatic		-	19.3 (7.2)	0–44	139 (16.3)
	Mood Disturbance		-	10.6 (4.4)	0–24	139 (16.3)
	Cognitive		-	4.9 (2.3)	0–12	139 (16.3)
	Anger		-	9.8 (4.4)	0–24	139 (16.3)
	Relationships		-	7.1 (2.9)	0–16	139 (16.3)
Functional Screener (Neurodisability)			-	172.7 (26.6)	60–240	0
Ex-Armed Forces			47 (5.5)	-	-	0
Traumatic Brain Injury			78 (9.2)	-	-	17 (2.0)
History of Substance Use			177 (20.8)	-	-	91 (10.7)
Age			-	31.6 (12.1)	18–76	0
Being held on remand			100 (11.7)	-	-	0
Homeless or marginally housed			285 (33.5)	-	-	2 (0.2)
Bullied			134 (15.7)	-	-	0
Excluded			487 (57.2)	-	-	0
History of Suicidality			102 (12.0)	-	-	33 (3.9)
History of Self-harm			93 (10.9)	-	-	38 (4.5)
History of both Suicide and Self-harm			70 (8.2)	-	-	48 (5.6)
Ethnicity		White	741 (87.0)	-	-	0
		Black or Black British	19 (2.2)	-	-	0
		Asian or Asian British	33 (3.9)	-	-	0
		Other	59 (6.9)	-	-	0

#### Missing data

48 individuals (5.6%) had missing data on the history of suicide and self-harm and these were removed listwise from the analysis. 139 individuals did not complete the mental health module. Module completion was at the discretion of prison staff, and it may be that there were time constraints, or these individuals were not deemed a priority to complete the mental health modules. T-tests and Chi-Squared tests of independence shown in Table 2 demonstrated that individuals with missing data compared to individuals without missing data included a higher proportion of homeless and were younger. Consequently, we removed those with missing data but note this as a limitation of this study. The final sample size for analysis was therefore 665 (16% of all new entrants over the eligible time period).

**Table 2:** Comparison of those who were included in the final analysis with those who were excluded for missing data.

Variable	Excluded from Analysis for Missing Data (n = 187)		Included in Final Analysis (n = 665)		Results of Chi-Squared test of independence or Welch’s t-test to compare means	
	N = (%)	Mean (SD)	N = (%)	Mean (SD)	Test statistic	P value
Functional Screener (Neurodisability)	-	160.8 (27.7)	-	160.2 (30.4)	t = 0.230	.818
Ex-Armed Forces	7 (3.7)	-	40 (6.0)	-	χ2 = 1.126	.289
Traumatic Brain Injury	12 (6.4)	-	66 (9.9)	-	χ2 = 1.902	.168
History of Substance Use	35 (18.7)	-	142 (21.4)	-	χ2 = 0.601	.438
Age	-	30.1 (8.5)	-	32.2 (12.3)	t = 2.728	.007**
Homeless or marginally housed	85 (45.5)	-	200 (30.1)	-	χ2 = 13.804	< .001***
Bullied	37 (19.7)	-	97 (14.6)	-	χ2 = 2.334	.127
Excluded	111 (59.4)	-	376 (56.5)	-	χ2 = 0.155	.694
History of Suicidality	23 (12.3)	-	79 (11.9)	-	χ2 = < .001	1.000
History of Self-harm	21 (11.2)	-	72 (10.8)	-	χ2 = < .001	1.000
History of both Suicide and Self-harm	15 (8.0)	-	55 (8.3)	-	χ2 = < .001	.989

Note

* = p is significant at 0.05

** = p is significant at 0.01

*** = p is significant at 0.001 Higher scores on the functional screener for neurodisability are indicative of more neurodisability/lower functional ability. Percentages are valid percentages, so account for missing data on any variable.

#### Comparison to wider prison population

We compared our sample demographics to the wider prison population [33], to assess the extent to which our results could be generalised to prisoners across the UK. The mean age of our sample was 31.6. The Ministry of Justice reports the percentage of prisoners which fall into an age range, and 30–39 is the modal age range (33% of prisoners). White prisoners make up 87% of our sample, but 72% of the prison population in England and Wales. This could be reflective of Wales having a higher proportion of White prisoners than England, as our sample is representative of the demographic profile of the specific prison in this study.

### Ethics

Ethical approval for this study was granted by the University of Exeter Department of Psychology research ethics committee (application ID: 492874), and the HMPPS (His Majesty’s Prison and Probation Service) National Research Committee. The prison gave permission to analyse this data, as the data controller. As part of the routine screening process, participants gave consent for their anonymised data being used for research purposes. Prisoners could choose not to consent to their data being used with no consequences for their treatment in prison, and could also choose not to answer any individual question (e.g. by selecting ‘prefer not to say’). Researchers had no access to information that could identify individual participants during or after data collection.

### Measures

The Do-IT Profiler [34] is a screening tool designed in collaboration with prison staff. It is used in prisons to understand background information about each prisoner’s vulnerabilities (e.g., substance use, mental health problems, experiences of education), and to understand strengths and difficulties that may be associated with neurodisability and may impact that prisoner’s engagement with education and rehabilitation. It is currently used in all prisons in Wales and Scotland, and several prisons in England. The Do-IT Profiler is a modular, computerised screening tool with built-in accessibility features, such as the option to have questions read aloud in different languages. A member of prison staff was available to supervise and assist with completion where required.

The modules and questionnaires included in the Do-IT Profiler are designed within a transdiagnostic framework. The philosophy of the screening tool is to reject arbitrary diagnostic thresholds (as would be used in medical models) and instead adopt a transdiagnostic functional needs assessment, to understand an individual’s strengths and weaknesses. The modules were developed collaboratively with forensic psychologists and prison education services to establish their practical efficacy in prison populations. This is important, as they are used to screen prisoners who typically have very complex, multifaceted profiles of need [34,35], and prisons need to understand their needs in practical terms to implement interventions. Data from the Do-IT Profiler has been used for research in other published studies of prison samples [36,37].

Prisoners completed the following modules (other modules are available to prison staff but were outside the scope of this research):

#### About me

This module collected self-reported demographic variables (including age), as well as information on whether the individual had ever been excluded from school, and whether they had ever been bullied whilst at school. They were also asked whether they had been in the armed forces. Prisoners were asked to describe their living situation, with the option of selecting from a dropdown menu of items (examples include ‘homeless’ and ‘living independently (with or without others)’). The researchers manually created a binary variable for prisoners being homeless or marginally housed, where 1 indicated being homeless or marginally housed and 0 indicated a stable living situation. We considered it appropriate to combine being homeless and marginally housed, because people in unstable living conditions are comparable to those who are literally homeless in their rates of poor mental health outcomes [38]. Examples of being marginally housed include ‘sofa-surfing’ or ‘staying with friends’. The presence of a possible traumatic brain injury was collected with the following question: ‘Have you ever had a significant injury to your head or face?’. History of suicide attempts and self-harm, as well as substance use problems, were collected with the following question ‘Do you have a history of any of the following… self-harming? attempted suicide? Substance misuse problems (e.g., illegal or legal drugs, alcohol)?’.

#### Functional Screener for Neurodisability

A 60-item questionnaire, with questions scored on a Likert-scale between 1 and 4. This is a transdiagnostic measure, which aims to capture functional, self-reported, strengths and difficulties often associated with neurodisability in four broad domains–cognitive, social, behavioural, and physical. Scores range between 60 and 240, with lower scores indicative of more functional disability. Example questions include ‘I tend to act impulsively, without thinking things through’ and ‘I have difficulty recognising other people’s emotions’.

#### How I feel

How I feel’ is a module assessing symptoms of mental health problems. It comprises 30 items, scored on a Likert scale from 0–4. The questionnaire was developed in collaboration with prisons, and so has not yet had the structure formally validated. We hypothesised a five-factor structure, with questions pertaining to psychosomatic symptoms (e.g., ‘experienced an increase in headaches’), mood disturbance (e.g., ‘experienced feeling depressed or low’), cognitive symptoms (e.g., ‘experienced difficulty maintaining concentration’), anger (e.g., ‘said something in anger then later regretted it’), and relationship difficulties (e.g., ‘Felt less inclined to make or keep close friendships or relationships’). Our confirmatory factor analysis (see S1 File) confirmed this factor structure (Comparative Fit Index = 0.992, where a CFI greater than 0.95 is considered a good fit [39]. Cronbach’s alpha values for each subscale were also acceptable, ranging from 0.717 (relationship difficulties) to 0.898 (anger).

## Results

### Descriptive statistics

As shown in Table 2, 12% of the sample self-reported a history of suicidality, 11% reported historic self-harm, and 8% reported both. 9.2% of the sample had experienced a traumatic brain injury, 21% reported problems with substance use, and 34% reported being homeless or marginally housed. 16% of prisoners reported being bullied when they were at school, and 57% had been excluded from school at least once.

**Fig 1:**
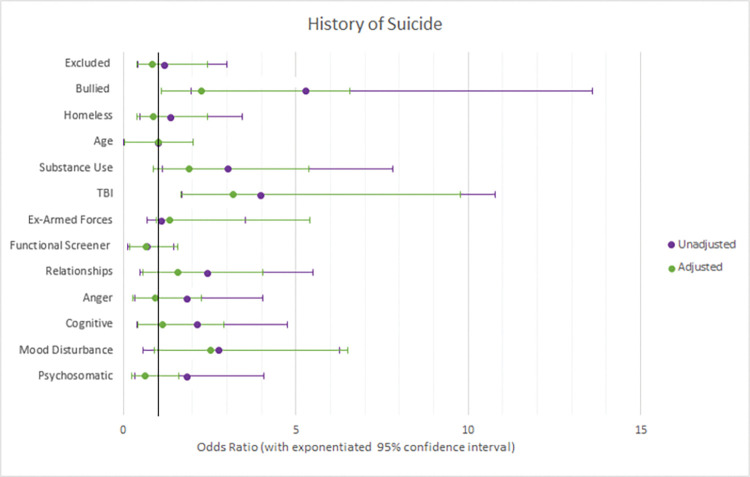
Forest plots of adjusted vs unadjusted odds ratios with 95% confidence intervals for factors associated with historic self-harm.

**Fig 2:**
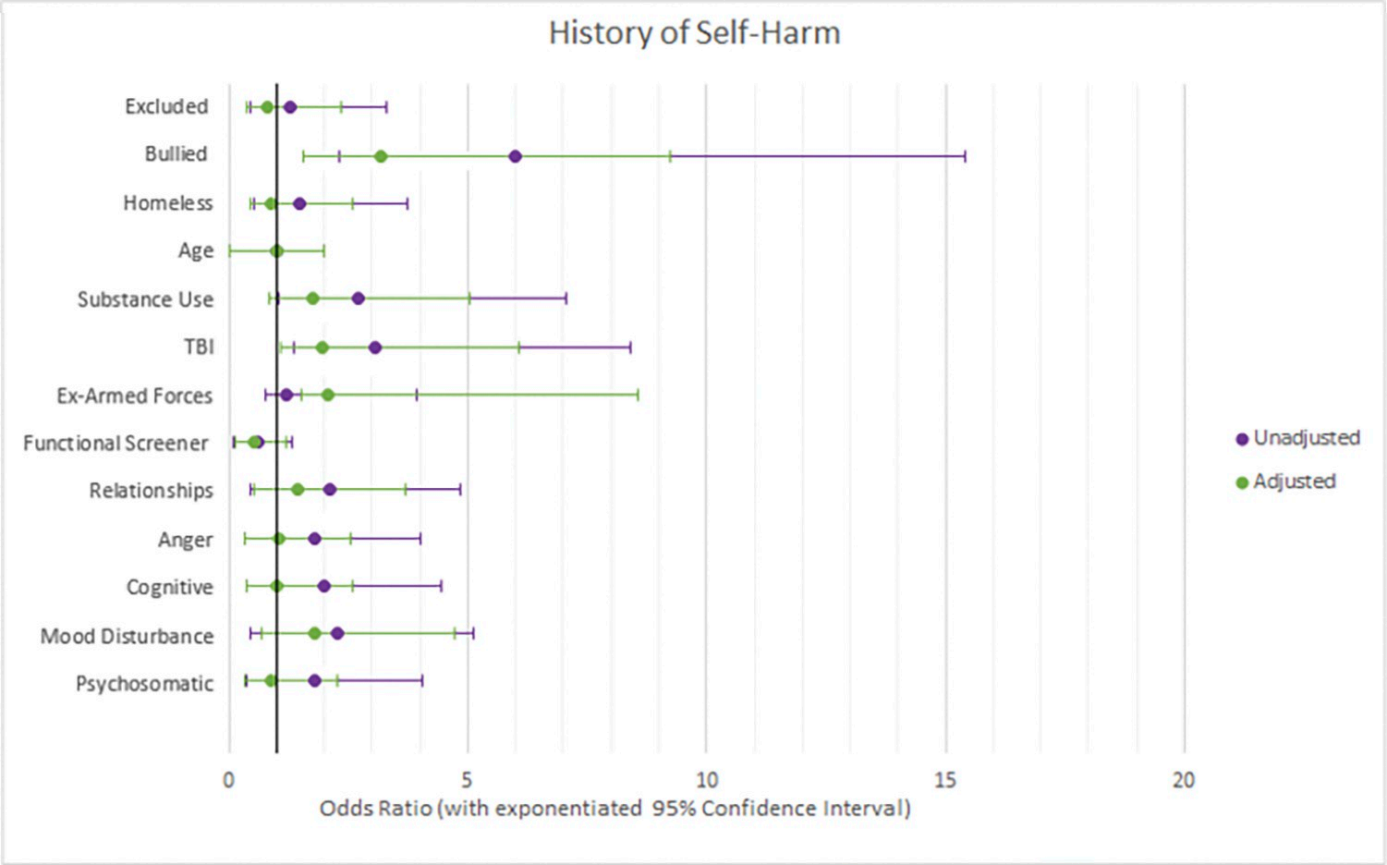
Forest plots of adjusted vs unadjusted odds ratios with 95% confidence intervals for factors associated with historic suicide.

**Fig 3:**
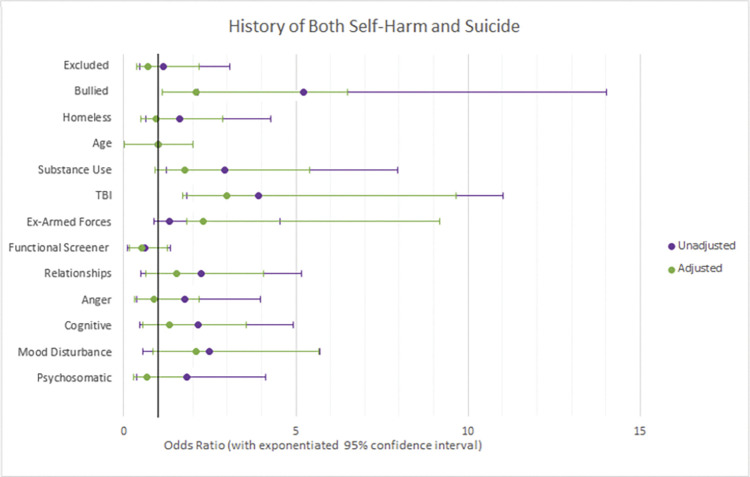
Forest plots of adjusted vs unadjusted odds ratios with 95% confidence intervals for factors associated with both historic self-harm and suicide.

Three logistic regression models were used to test which variables were associated with having a history of self-harm, suicide, or both. The results of these models are reported in Table 3, with adjusted and unadjusted odds ratios reported in Figs 1–3, with exponentiated 95% confidence intervals. Adjusted odds ratios were adjusted for all 13 variables.

**Table 3:** Results of three logistic regression models, with outcome variables: Having a history of suicide, self-harm, and both.

Variable		Adjusted Odds Ratio			P Value			Exponentiated 95% Confidence Intervals		
		Suicide	Self-Harm	Both	Suicide	Self-Harm	Both	Suicide	Self-Harm	Both
Mental Health Symptoms (Z-Scores)	Psychosomatic	0.617	0.856	0.645	.040*	.510	.107	0.392–0.979	0.546–1.384	0.394–1.142
	Mood Disturbance	2.578	1.827	2.171	**< .001** ***	.013*	**.004** **	1.638–3.965	1.132–2.902	1.264–3.561
	Cognitive	1.041	0.915	1.197	.863	.713	.511	0.726–1.777	0.628–1.577	0.787–2.233
	Anger	0.892	1.008	0.849	.536	.967	.439	0.641–1.324	0.705–1.511	0.570–1.325
	Relationships	1.575	1.400	1.509	.043*	.162	.123	1.015–2.455	0.878–2.269	0.887–2.545
Functional Screener (Neurodisability) (Z-Score)		0.670	0.589	0.581	**.003** **	**< .001** ***	**< .001** ***	0.479–0.923	0.362–0.708	0.354–0.747
Ex-Armed Forces		1.361	1.938	1.907	.602	.270	.329	0.388–4.045	0.584–6.472	0.489–6.870
Traumatic Brain Injury		3.333	2.105	3.257	**.001** **	.059	**.006** **	1.511–6.601	0.872–4.122	1.284–6.683
History of Substance Use		1.936	1.796	1.864	.037*	.074	.089	1.016–3.495	0.914–3.291	0.861–3.625
Age		1.004	0.987	0.989	.747	.355	.460	0.976–1.026	0.956–1.010	0.955–1.014
Homeless or marginally housed		0.822	0.863	0.916	.538	.648	.810	0.454–1.571	0.471–1.676	0.457–1.909
Bullied		1.964	2.726	1.750	.051	**.003** **	.158	1.155–4.318	1.625–6.087	0.965–4.401
Excluded		0.817	0.777	0.692	.542	.464	.334	0.437–1.604	0.400–1.550	0.333–1.494

Note

* = p is significant at 0.05

** = p is significant at 0.01

*** = p is significant at 0.001. Odds Ratios are adjusted for all 13 variables. To correct for multiple comparisons, because of using three models for our three outcome variables, we calculated the Bonferroni alpha value as 0.017. P values in **bold** remain statistically significant when correcting for multiple comparisons.

### Risk factors associated with history of attempted suicide

Prisoners who reported a historic TBI were at 3.3 times increased odds of reporting a history of attempted suicide than those who did not (95% CI: 1.51–6.60). Reporting substance use was also significantly associated with historic suicide attempts (OR: 1.9, 95% CI: 1.02–3.50). Furthermore, each one standard deviation increase in scores on the functional screener (where higher scores indicate less neurodisability) was associated with decreased odds of having a historic suicide attempt by an odds ratio of 0.7 (95% CI: 0.48–0.92).

Three dimensions of the mental health questionnaire were associated with historic attempted suicide. A one standard deviation increase in scores on the mood disturbance subscale significantly increased odds of reporting a historic suicide attempt with an odds ratio of 2.6 (95% CI: 1.64–3.97), and a one standard deviation increase in reported relationship difficulties increased odds by a ratio of 1.6 (95% CI: 1.02–2.46). A one standard deviation in psychosomatic symptoms reduced odds of reporting historic suicide attempts (OR: 0.6, 95% CI: 0.39–0.98).

### Risk factors associated with historic self-harm

There were three risk factors associated with reporting historic self-harm. Firstly, those who were bullied at school had 2.7 times increased odds of reporting self-harm (95% CI: 1.63–6.09). A one standard deviation increase in scores on the functional screener was associated again with decreased odds of reporting historic self-harm by an odds ratio of 0.6 (95% CI: 0.36–0.71). Finally, a one standard deviation increase in score for mood disturbance multiplied the odds for reporting historic self-harm by 1.8 (95% CI: 1.13–2.90).

### Risk factors associated with history of both suicidality and self-harm

Reporting a TBI multiplied odds of having *both* historic self-harm and suicide attempts by 3.3 (95% CI: 1.28–6.68). Individuals who scored lower on the functional screener for neurodisability were also at increased odds of reporting both historic self-harm and suicide attempts (OR: 0.6 for a 1SD increase in score, where higher scores indicate less neurodisability, 95% CI: 0.35–0.75), and individuals who scored higher on the mood disturbance subscale also had increased odds of reporting both historic suicide and self-harm (OR: 2.2 for a 1SD increase in score, where higher scores indicate more mood disturbance, 95% CI: 1.26–3.56).

## Discussion

We found unique factors associated with suicide and self-harm. We considered adjusted odds ratios here, and consequently, the risk conferred by each factor is unique of the others. The unique risk factors associated with a history of attempted suicide were self-reporting a TBI, and reporting substance use problems. For self-harm, the unique risk factor was reporting having been bullied at school. The risk factors associated with having a history of both self-harm and suicide were TBI, scores indicating more functional disability on the screener which captured disability in cognitive, social, behavioural, and physical domains, and indicating more mood disturbance on the mental health measure. Mental health measures were adjusted for in all models, so TBI, substance use, being bullied at school, and functional disability uniquely confer risk even whilst controlling for mood disturbance, anger, relationship difficulties, psychosomatic symptoms, and cognitive symptoms.

The findings exemplify the need to examine and consider risk factors for suicide attempt, self-harm, and both histories individually. While both histories share common risk factors (i.e., mood disturbance, traumatic brain injury exposure, and neurodisability); there are unique associations of multiple vulnerabilities for each behaviour or combination of behaviours highlighting unique matrices of vulnerability. Unique vulnerabilities imply different treatment approaches may be needed. The significance of TBI, functional neurodisability, and mood disturbance for combined histories of self-harm and suicide attempts may hold relevance for risk management and intervention approaches in prison settings as adjustments to existing interventions may be required, to avoid removal of support due to behaviour [31]. Moreover, the findings highlight the need to utilise research to further investigate prisoners with enhanced vulnerabilities such as those indicated by the functional neurodisability screener to improve mental health prevention and intervention efforts in the prison system [40].

Additionally, though the administrative data used in the current study are older (2014–2016), the findings reflect the ongoing issues in identifying and supporting prisoners who have multiple vulnerabilities [41]. A recent review of prison mental health care in England notes that currently, there are still a paucity of mental health programmes for prisoners in England and Wales which are accessible and able to support prisoners with complex or multiple vulnerabilities. There are specific services available such as the Offender Personality Disorder Programme (OPD), drug and alcohol services, and various psychological therapies (e.g., Cognitive Behavioural Therapy [CBT]). However, each of these services are not equipped to address more complex profiles of vulnerabilities, of which a large proportion of the prison population present with [42]. Moreover, many services are supplemented by voluntary and community sector (VCS) organisations such as ‘Rethink Mental Illness’ which impacts the accessibility of mental health services as their availability varies on a regional basis. Finally, many services are inaccessible for individuals who present as neurodivergent, indicating a further barrier to support. Overall, the current study findings support the need for service development, and staff training programmes to address the rising needs of prisoners with multiple vulnerabilities.

Finally, the current study findings support the roll-out of screening tools which assess a broader range of vulnerabilities rather than a narrow set of risk factors [41]. Broad screening tools including questions on physical and neurodiverse issues, which the Do-IT profiler covers, align with NICE (National Institute for Health and Care Excellence) guidance for first stage health assessments upon reception to prison [43]. Such tools allow for comprehensive screening of prisoners upon entry to prison, which directs the level of service need they may require during their sentence. This is particularly important during the first few weeks in prison, where risk of suicide is elevated [19]. Moreover, gaining an understanding of the broad profile of vulnerabilities a prisoner presents with in the first instance allows for risk management procedures to be implemented immediately, rather than relying on secondary screening, which may take place up to a week following reception [44]. Adequate programmes addressing adaptations to existing services to accommodate broader neuro-divergences are still lacking, thus present an avenue for future research.

### Limitations

A fundamental limitation of this study is the cross-sectional nature of the data. This severely limits the ability to draw causal inference from the findings. We lacked a follow-up outcome of actual suicide and self-harm in prison, so future research should look to include these to determine the actual utility of screening for the factors we present here on entry to prison. The prison environment significantly differs from the community, so different risk factors may be more salient inside prison.

The self-report nature of these measures also presents a limitation. Self-report measures are subjective by their nature and focus on the most salient problems for the individual at the time. They also rely on insight and choice to report. Under-reporting may have occurred, particularly for self-harm, suicide, and substance use, as prisoners may have anticipated stigma or punishment from officers. Prisoners may lack insight into the presence of a brain injury. However, Schofield and colleagues [45] found that prisoners are reliable survey respondents of self-reported traumatic brain injury by comparing their survey responses to hospital records.

We also found missingness in the data, which could have weakened associations. 16% of individuals who completed the screening did not complete the mental health questionnaires. Whilst we did establish that those who were excluded from the analysis for missing data on the mental health questionnaires or the outcome variables did not significantly differ on any variables aside from age, and the proportion who were homeless or marginally housed, this could be a source of bias in the results. Specifically, it could explain why being homeless was not associated with any increased risk as a greater proportion of those who were homeless were missing. It is also possible that our resultant reduced sample size was underpowered to detect an effect, generating type 2 errors, given that we included 13 variables in each model and our outcome variables were relatively rare. Whilst we did test for multicollinearity before running our models, we note that some overlapping constructs may be being captured here–for example, between the presence of a TBI and our measure of functional neurodisability. Further work is required to disentangle the roles of these factors.

Several salient established risk factors associated with self-harm and suicidality were not collected as part of the screening process, including psychiatric diagnoses (e.g. of personality disorders), and histories of trauma [46,47]. It is possible here that our measured variable of being bullied at school is acting as a proxy for experiences of trauma or victimisation. This is a significant limitation, as these factors could be more important in explaining vulnerability to suicidality and self-harm than the variables collected here. The available data on ethnicity was also very limited, with very coarse categories and many prisoners being described as ‘other’. We therefore didn’t include race in this analysis but note that studies have shown that rates of self-harm vary by ethnic group, with White prisoners having higher rates of self-harm in the UK [2] and this is therefore an important factor for inclusion in future work. Additionally, our findings are from a male prison and it is known that rates of self-harm and suicide are higher in women’s prisons [2,8]. Further work is needed to establish whether the risk factors identified here are also salient in the women’s secure estate.

We therefore don’t advocate for the factors discussed here to be collected in place of established risk factors, but instead highlight the importance of expanding prison’s understanding of the ‘whole-person’ in the context of their biological, psychological, and social risk factors. If a prisoner is engaging with a treatment pathway for a psychiatric diagnosis, identifying the presence of, for example, comorbid neurodisability could help with making sure treatment sessions are accessible and not contraindicated by functional difficulties.

### Implications and future directions

Longitudinal research is needed to establish whether the vulnerability factors we identify here as being associated with reporting historic self-harm and suicide on entry to prison are also associated with actual self-harm and suicide whilst in prison. If such longitudinal research corroborates our findings prisons should utilise screening tools which assess for functional disability, traumatic brain injury, being bullied at school, substance use, and mood disturbance. There is also a need to determine whether these risk factors are additive and multiplicative, but this requires larger sample cross-sectional work.

This study provides evidence indicating that multidisciplinary care pathways, equipped to treat multimorbidity across TBI, mental health, and substance use problems, are essential [48]. Mental health resources in prisons are inadequate for the level of need, and there is a risk that without multidisciplinary approaches which are equipped to treat complex profiles (which we see in this study) treatment could be ineffective. Instead, adaptations to treatment plans are necessary to account for the presence of multimorbidity, particularly with high levels of symptom overlap [49]. For example, fatigue and headaches could be symptoms of both depression and TBI, and CBT interventions which use techniques such as behavioural intervention may need to accommodate this [49].

Additionally, embedding an understanding of complex ‘external explanations’ for self-harm and suicide amongst prison officers could promote more compassionate responses. Gill and colleagues argue that compassion is evoked when negative behaviour from an outgroup is explained by enduring causal forces (such as traumatic brain injury), which elucidate perceived suffering as a result of factors outside of the individual’s control [50]. A better understanding of prisoners complex multimorbidity could therefore increase compassion, and in turn reduce emotional burnout in prison staff [51].

At the point of entry to prison, screening should occur promptly, and account for a broad range of vulnerability factors to inform treatment and risk management protocols in prisons. If screening is appropriately utilised to allocate support for prisoners at risk of suicide and self-harm is improved, traumatic exposure for staff may be reduced too.

## Supporting information

S1 Checklist Checklist of items to be included in reports of observational studies. (DOCX)

S1 File **S1 Table:** Results of the confirmatory factor analysis (CFA) for the subscales of the mental health questionnaire. Comparative Fit Index (CFI) for the model was 0.992, robust CFI was 0.944. A Diagonally Weighted Least Squares Estimator was used as this data comprises Likert responses (ordinal data). **S2 Table:** Cronbach’s alpha reliability scores for each subscale of the mental health questionnaire.
(DOCX)
