# Supplementary material for: Profiles of vulnerability for suicide and self-harm in UK prisoners: Neurodisability, mood disturbance, substance use, and bullying
Source: PLoS One. 2024 Jan 3;19(1):e0296078. doi: 10.1371/journal.pone.0296078 (PMC10763929; doi:10.1371/journal.pone.0296078)
Supplement: S1 Checklist — (DOCX) [file pone.0296078.s001.docx]

STROBE Statement—checklist of items that should be included in reports of observational studies

|  | Item No. | Recommendation | Page  No. | Relevant text from manuscript |
| --- | --- | --- | --- | --- |
| **Title and abstract** | 1 | (*a*) Indicate the study’s design with a commonly used term in the title or the abstract | 2 | Administrative screening data |
|  |  | (*b*) Provide in the abstract an informative and balanced summary of what was done and what was found | 2 | See abstract |
| Introduction | | | |  |
| Background/rationale | 2 | Explain the scientific background and rationale for the investigation being reported | 3-5 | See ‘Introduction’ section |
| Objectives | 3 | State specific objectives, including any prespecified hypotheses | 6 | Section entitled ‘The Current Study’ |
| Methods | | | |  |
| Study design | 4 | Present key elements of study design early in the paper | 3, 6-9 | Study design first introduced on page 3 in the first paragraph, with more detail in pages 6-9 |
| Setting | 5 | Describe the setting, locations, and relevant dates, including periods of recruitment, exposure, follow-up, and data collection | 6-9 | Detailed methods section provided on pages 6-9 |
| Participants | 6 | (*a*) *Cohort study*—Give the eligibility criteria, and the sources and methods of selection of participants. Describe methods of follow-up  *Case-control study*—Give the eligibility criteria, and the sources and methods of case ascertainment and control selection. Give the rationale for the choice of cases and controls  *Cross-sectional study*—Give the eligibility criteria, and the sources and methods of selection of participants | 6 | The study examined administrative data from 852 adult male prisoners screened on entry to HMP Parc (a prison in Wales, UK) between 2014 and 2016. 100 of these were being held on remand, meaning that they were being held pre-trial and had not been formally convicted of a crime at the time of screening, and 752 had been convicted. Individuals were screened at induction to prison, with the option for people to decline if they did not wish to complete the screening. |
|  |  | (*b*) *Cohort study*—For matched studies, give matching criteria and number of exposed and unexposed  *Case-control study*—For matched studies, give matching criteria and the number of controls per case | NA |  |
| Variables | 7 | Clearly define all outcomes, exposures, predictors, potential confounders, and effect modifiers. Give diagnostic criteria, if applicable | 3-5, 7-9 | A full definition of all variables and their operationalisation is provided in the introduction and methods sections |
| Data sources/ measurement | 8* | For each variable of interest, give sources of data and details of methods of assessment (measurement). Describe comparability of assessment methods if there is more than one group | ­7-9 | A full description of all variables and their operationalisation is provided in the methods section |
| Bias | 9 | Describe any efforts to address potential sources of bias | 7 | 48 individuals (5.6%) had missing data on the history of suicide and self-harm and these were removed listwise from the analysis. 139 individuals did not complete the mental health module. Module completion was at the discretion of prison staff, and it may be that there were time constraints, or these individuals were not deemed a priority to complete the mental health modules. T-tests and Chi-Squared tests of independence shown in Table 2 demonstrated that non-completers of the mental health questionnaire compared to completers included a higher proportion of homeless and were younger. |
| Study size | 10 | Explain how the study size was arrived at | 6 | The study examined administrative data from 852 adult male prisoners screened on entry to HMP Parc (a prison in Wales, UK) between 2014 and 2016. |

Continued on next page

| Quantitative variables | 11 | Explain how quantitative variables were handled in the analyses. If applicable, describe which groupings were chosen and why | 7-9 | Operationalisation of quantitative variables and their handling in the analysis is reported in the methods section. |
| --- | --- | --- | --- | --- |
| Statistical methods | 12 | (*a*) Describe all statistical methods, including those used to control for confounding | 12 | Three logistic regression models were used to test which variables were associated with having a history of self-harm, suicide, or both. The results of these models are reported in Table 3, with adjusted and unadjusted odds ratios reported in Figures 1-3, with 95% confidence intervals. |
|  |  | (*b*) Describe any methods used to examine subgroups and interactions | NA |  |
|  |  | (*c*) Explain how missing data were addressed | 7 | 48 individuals (5.6%) had missing data on the history of suicide and self-harm and these were removed listwise from the analysis. 139 individuals did not complete the mental health module. Module completion was at the discretion of prison staff, and it may be that there were time constraints, or these individuals were not deemed a priority to complete the mental health modules. T-tests and Chi-Squared tests of independence shown in Table 2 demonstrated that non-completers of the mental health questionnaire compared to completers included a higher proportion of homeless and were younger. |
|  |  | (*d*) *Cohort study*—If applicable, explain how loss to follow-up was addressed  *Case-control study*—If applicable, explain how matching of cases and controls was addressed  *Cross-sectional study*—If applicable, describe analytical methods taking account of sampling strategy | NA |  |
|  |  | (*e*) Describe any sensitivity analyses | NA |  |
| Results | | | | |
| Participants | 13* | (a) Report numbers of individuals at each stage of study—eg numbers potentially eligible, examined for eligibility, confirmed eligible, included in the study, completing follow-up, and analysed | 6, 7 | The study examined administrative data from 852 adult male prisoners.  48 individuals (5.6%) had missing data on the history of suicide and self-harm and these were removed listwise from the analysis. 139 individuals did not complete the mental health module. Module completion was at the discretion of prison staff, and it may be that there were time constraints, or these individuals were not deemed a priority to complete the mental health modules. T-tests and Chi-Squared tests of independence shown in Table 2 demonstrated that non-completers of the mental health questionnaire compared to completers included a higher proportion of homeless and were younger. Consequently, we removed the 139 non-completers of the mental health module but note this as a limitation of this study. The final sample size for analysis was therefore 665. |
|  |  | (b) Give reasons for non-participation at each stage | 6, 7 | As above. |
|  |  | (c) Consider use of a flow diagram | NA |  |
| Descriptive data | 14* | (a) Give characteristics of study participants (eg demographic, clinical, social) and information on exposures and potential confounders | 10 | This information is provided in Table 1 |
|  |  | (b) Indicate number of participants with missing data for each variable of interest | 11 | This information is provided in Table 2 |
|  |  | (c) *Cohort study*—Summarise follow-up time (eg, average and total amount) | NA |  |
| Outcome data | 15* | *Cohort study*—Report numbers of outcome events or summary measures over time | NA |  |
|  |  | *Case-control study—*Report numbers in each exposure category, or summary measures of exposure | NA |  |
|  |  | *Cross-sectional study—*Report numbers of outcome events or summary measures | 12 | As shown in Table 2, 12% of the sample self-reported a history of suicidality, 11% reported historic self-harm, and 8% reported both. 9.2% of the sample had experienced a traumatic brain injury, 21% reported problems with substance use, and 34% reported being homeless or marginally housed. 16% of prisoners reported being bullied when they were at school, and 57% had been excluded from school at least once. |
| Main results | 16 | (*a*) Give unadjusted estimates and, if applicable, confounder-adjusted estimates and their precision (eg, 95% confidence interval). Make clear which confounders were adjusted for and why they were included | 14, 15 | Adjusted models are reported in table 3, whilst adjusted and unadjusted coefficients with 95% confidence intervals are reported in figures 1, 2, and 3 |
|  |  | (*b*) Report category boundaries when continuous variables were categorized | NA |  |
|  |  | (*c*) If relevant, consider translating estimates of relative risk into absolute risk for a meaningful time period | NA |  |

Continued on next page

| Other analyses | 17 | Report other analyses done—eg analyses of subgroups and interactions, and sensitivity analyses | Statistical Appendix | The statistical appendix reports on a Confirmatory Factor Analysis and Cronbach’s alpha values calculated for the mood disturbance subscales. |
| --- | --- | --- | --- | --- |
| Discussion | | | | |
| Key results | 18 | Summarise key results with reference to study objectives | 16 | We found unique factors associated with suicide and self-harm. We considered adjusted odds ratios here, and consequently, the risk conferred by each factor is unique of the others. The unique risk factors associated with a history of attempted suicide were self-reporting a TBI, and reporting substance use problems. For self-harm, the unique risk factor was reporting having been bullied at school. The risk factors associated with having both self-harm and suicide were TBI, scores indicating more functional disability on the screener which captured disability in cognitive, social, behavioural, and physical domains, and indicating more mood disturbance on the mental health measure. |
| Limitations | 19 | Discuss limitations of the study, taking into account sources of potential bias or imprecision. Discuss both direction and magnitude of any potential bias | 16-17 | The self-report nature of these measures presents a limitation. Self-report measures are subjective by their nature and focus on the most salient problems for the individual at the time. They also rely on insight and choice to report. Under-reporting may have occurred, particularly for self-harm, suicide, and substance use, as prisoners may have anticipated stigma or punishment from officers. However, Schofield and colleagues (46) found that prisoners are reliable survey respondents of self-reported traumatic brain injury by comparing their survey responses to hospital records. We also found missingness in the data, which could have weakened associations. 16% of individuals who completed the screening did not complete the mental health questionnaires. Whilst we did establish that those with missing data on the mental health questionnaire did not significantly differ on any variables aside from age, and the proportion who were homeless or marginally housed, this could be a source of bias in the results. Specifically, it could explain why being homeless was not associated with any increased risk as a greater proportion of those who were homeless were missing. |
| Interpretation | 20 | Give a cautious overall interpretation of results considering objectives, limitations, multiplicity of analyses, results from similar studies, and other relevant evidence | 16-17 | See discussion section |
| Generalisability | 21 | Discuss the generalisability (external validity) of the study results | 17 | Longitudinal research is needed to establish whether the vulnerability factors we identify here as being associated with reporting historic self-harm and suicide on entry to prison are also associated with actual self-harm and suicide whilst in prison. If such longitudinal research corroborates our findings prisons should utilise screening tools which assess for functional disability, traumatic brain injury, being bullied at school, substance use, and mood disturbance. Prisons should prioritise allocating mental health resource during the first weeks in prison to those with the risk factors identified in this study. There is also a need to determine whether these risk factors are additive and multiplicative, but this requires larger sample cross-sectional work. Prevention is undoubtedly better than cure, and public health interventions to reduce incidence of TBI, and to reduce bullying in schools, are supported by our results. However, at the point of entry to prison, screening should occur promptly, and account for static and dynamic risk factors. If screening is appropriately utilised to allocate support for prisoners at risk of suicide and self-harm is improved, traumatic exposure for staff will be reduced too. Additionally, disseminating these results to prison stakeholders will help them to better characterise the broader pattern of risk, and promote compassionate responses to suicidality and self-harm in prisons. |
| Other information | |  | | |
| Funding | 22 | Give the source of funding and the role of the funders for the present study and, if applicable, for the original study on which the present article is based | 1 (title page) | This project was funded by HMP/YOI Parc. Hope Kent was supported by the Economic and Social Research Council (grant number ES/P000630/1). The funders had no role in the study design, analysis, and interpretation of the data, the writing of the report, or the decision to submit the article for publication. |

*Give information separately for cases and controls in case-control studies and, if applicable, for exposed and unexposed groups in cohort and cross-sectional studies.

**Note:** An Explanation and Elaboration article discusses each checklist item and gives methodological background and published examples of transparent reporting. The STROBE checklist is best used in conjunction with this article (freely available on the Web sites of PLoS Medicine at http://www.plosmedicine.org/, Annals of Internal Medicine at http://www.annals.org/, and Epidemiology at http://www.epidem.com/). Information on the STROBE Initiative is available at www.strobe-statement.org.
