# Supplementary material for: Profiles of vulnerability for suicide and self-harm in UK prisoners: Neurodisability, mood disturbance, substance use, and bullying
Source: PLoS One. 2024 Jan 3;19(1):e0296078. doi: 10.1371/journal.pone.0296078 (PMC10763929; doi:10.1371/journal.pone.0296078)
Supplement: S1 File — S1 Table: Results of the confirmatory factor analysis (CFA) for the subscales of the mental health questionnaire. Comparative Fit Index (CFI) for the model was 0.992, robust CFI was 0.944. A Diagonally Weighted Least Squares Estimator was used as this data comprises Likert responses (ordinal data). S2 Table: Cronbach’s alpha reliability scores for each subscale of the mental health questionnaire. (DOCX) [file pone.0296078.s002.docx]

**Statistical Appendix**

**Psychometric properties of, and questions in, the mental health questionnaire**

**TABLE S1.** Results of the confirmatory factor analysis (CFA) for the subscales of the mental health questionnaire. Comparative Fit Index (CFI) for the model was 0.992, robust CFI was 0.944. A Diagonally Weighted Least Squares Estimator was used as this data comprises Likert responses (ordinal data).

| Subscales/Items | Estimate | Std. Error | z – value | P value |
| --- | --- | --- | --- | --- |
| **Somatic symptoms**: |  |  |  |  |
| Q1: Experienced an increase in headaches | 1.000 |  |  |  |
| Q2: Experienced backache/joint pains | 1.113 | 0.063 | 17.716 | 0.000 |
| Q3: Experienced new or worsening skin problems | 0.927 | 0.072 | 12.860 | 0.000 |
| Q4: Experienced cramps, muscle pains or aches | 1.234 | 0.070 | 17.745 | 0.000 |
| Q5: Experienced dizziness or palpitations | 1.345 | 0.070 | 19.241 | 0.000 |
| Q6: Experienced chest pains | 1.201 | 0.071 | 16.842 | 0.000 |
| Q7: Experienced an increase in sweating | 1.271 | 0.070 | 18.095 | 0.000 |
| Q8: Experienced an increase in nervous twitches or habits such as nail biting | 1.376 | 0.073 | 18.810 | 0.000 |
| Q9: Experienced decrease in mobility | 1.341 | 0.076 | 17.665 | 0.000 |
| Q11: Experienced change in appetite | 1.326 | 0.076 | 17.441 | 0.000 |
| Q12: Experienced alteration of sleep pattern | 1.385 | 0.078 | 17.859 | 0.000 |
| **Mood disturbance**: |  |  |  |  |
| Q15: Felt unable to enjoy life | 1.000 |  |  |  |
| Q16: Experienced feeling depressed or low | 1.071 | 0.024 | 45.278 | 0.000 |
| Q17: Experienced crying for no apparent reason | 0.959 | 0.033 | 28.953 | 0.000 |
| Q18: Felt anxious, irritable or more angry with others | 1.082 | 0.025 | 42.997 | 0.000 |
| Q20: Experienced the death of someone in last 6 months | 0.587 | 0.050 | 11.674 | 0.000 |
| **Cognitive symptoms**: |  |  |  |  |
| Q10: Felt overwhelmed or made poor judgements | 1.000 |  |  |  |
| Q13: Experienced difficulty maintaining concentration | 1.035 | 0.028 | 37.470 | 0.000 |
| Q19: Experienced memory problems | 1.009 | 0.030 | 33.395 | 0.000 |
| **Anger/aggression**: |  |  |  |  |
| Q22: Experienced getting angry easily | 1.000 |  |  |  |
| Q23: Became angry due to things said or done in the past | 0.973 | 0.018 | 53.139 | 0.000 |
| Q24: Been in trouble due to temper | 0.959 | 0.016 | 58.336 | 0.000 |
| Q25: Said something in anger then later regretted | 0.942 | 0.021 | 45.179 | 0.000 |
| Q27: Frightened people by actions when angry | 0.926 | 0.022 | 41.294 | 0.000 |
| Q28: Used food/cigarettes for comfort when angry, frustrated or hurt | 0.886 | 0.025 | 35.436 | 0.000 |
| **Relationship difficulties**: |  |  |  |  |
| Q14: Less inclined to make/keep close friendships and relationships | 1.000 |  |  |  |
| Q21: Difficulties with relationships | 0.593 | 0.071 | 8.324 | 0.000 |
| Q29: Found it hard to tell others about feelings | 1.093 | 0.043 | 25.681 | 0.000 |
| Q30: Been upset but tried not to show others | 1.110 | 0.042 | 26.563 | 0.000 |

*Note: For the first factors in each category (Question 1, 15, 13, 22 and 14) no output values are provided as all other loadings are scaled according to them.*

**TABLE S2.** Cronbach’s alpha reliability scores for each subscale of the mental health questionnaire.

| **Subscale** | **Number of Items** | **α** |
| --- | --- | --- |
| Somatic symptoms | 11 | 0.882 |
| Mood disturbance | 6 | 0.817 |
| Cognitive symptoms | 3 | 0.787 |
| Anger | 6 | 0.898 |
| Relationship difficulties | 4 | 0.717 |
